# Copro-Molecular Identification of Tapeworms in Introduced Invasive Carnivores in Poland

**DOI:** 10.3390/pathogens11020110

**Published:** 2022-01-18

**Authors:** Katarzyna Buńkowska-Gawlik, Joanna Hildebrand, Marcin Popiołek, Dorota Merta, Agnieszka Perec-Matysiak

**Affiliations:** 1 Department of Parasitology, Faculty of Biological Sciences, University of Wrocław, 51-148 Wrocław, Poland; joanna.hildebrand@uwr.edu.pl (J.H.); marcin.popiolek@uwr.edu.pl (M.P.); agnieszka.perec-matysiak@uwr.edu.pl (A.P.-M.); 2 Department of Ecology and Environmental Protection, Institute of Biology, Pedagogical University of Kraków, 30-084 Kraków, Poland; dorota.merta@up.krakow.pl

**Keywords:** raccoon, raccoon dog, cestodes, copro-PCR

## Abstract

The raccoon (*Procyon lotor*) and the raccoon dog (*Nyctereutes procyonoides*) were introduced to Europe and, in the past decades, their populations have increased and adapted to synanthropic environments across Europe. In view of their possible further spread in Europe, the invasive species should be examined as potential reservoirs for helminths, including tapeworms. This study aims to investigate the prevalence and diversity of tapeworms in introduced wild carnivores in Poland by identifying cestode species based on copro-DNA analysis. A total of 214 individual fecal samples from non-native invasive carnivores, i.e., raccoons and raccoon dogs, and additionally 47 samples from native carnivores, i.e., European badgers (*Meles meles*), were analyzed for the presence of cestodes. PCR analysis of fecal samples targeting a fragment of mitochondrial (mt) 12S rRNA gene revealed the presence of cestode DNA in 19 of 103 (18.4%) raccoons, in 13 of 111 (11.7%) raccoon dogs and in 23 of 47 (48.9%) badgers. Sequence analysis demonstrated the presence of *Mesocestoides litteratus* in raccoons and raccoon dogs, while *Mesocestoides lineatus* was identified only in two samples derived from raccoon dogs. Moreover, in this study, *Atriotaenia incisa* was for the first time molecularly characterized by using fragments of mt 12S rRNA gene, and the DNA of this cestode species was detected in the fecal samples of all the examined host species.

## 1. Introduction

The raccoon (*Procyon lotor*) and the raccoon dog (*Nyctereutes procyonoides*) were introduced to Europe, mainly for fur farming and hunting reasons [1,2]. *Procyon lotor* is native to North and Central America and currently, the range of the raccoon population in Europe has extended to the west, east and south of the invasion core [1]. *Nyctereutes procyonoides* is endemic to East Asia. In the early twentieth century, it was introduced into western parts of Russia, from where it slowly expanded into Scandinavia, the Baltic states, and, by the 1960s, Poland and Germany [2]. Raccoons and raccoon dogs can be found in 20 and 33 countries in Europe, respectively, and their geographical ranges overlap to a large extent [2]. Both species are now listed in Europe as an invasive species of Union concern (Regulation (EU) No. 1143/2014), and member states are required to control pathways of introductions and manage established populations [3]. The raccoons’ and raccoon dogs’ habitats overlap with the range of other carnivores, e.g., badgers. 

Raccoons and raccoon dogs harbor a broad range of parasites and pathogens, of which several are of zoonotic importance, potentially causing serious health problems to humans or their companion animals [4,5,6,7,8,9,10]. Raccoons and raccoon dogs can be definitive hosts of cestodes. Tapeworms are a highly diversified group of parasites that can be found in a wide variety of hosts and can cause serious damage to wild and domestic animals, as well as human health. The most important among these is *Echinococcus multilocularis*, which causes human alveolar echinococcosis, considered as one of the most pathogenic zoonoses in the temperate and arctic regions of Europe, characterized by a long incubation period and a high fatality rate [11,12]. As compared to the red fox, less attention has been paid to the raccoon dog as a potentially important host for this cestode species. The first case of *E. multilocularis* in naturally infected raccoon dogs in Europe was noted in 2001 in Germany [13]. Thereafter, this cestode species was recorded in raccoon dogs from other European countries, such as Lithuania, Latvia, Slovakia, Sweden, Denmark, Estonia and Netherlands [12,14,15,16]. Relatively recent research from Germany [17] and Estonia [18] indicates the increasing importance of the raccoon dogs as definitive hosts, particularly as they are becoming widespread, have well-established populations and share the same areas with foxes. However, on the other hand, the role of *N. procyonoides* in the life cycle of *E. multilocularis* is still questionable because of its lower prevalence and abundance within natural infection, possibly because rodents are less common in their diet than in the diet of foxes [15]. To the best of our knowledge, in Poland this parasite was recorded in raccoon dogs only twice, in 2002 and 2003 [19,20]; therefore, at present, knowledge concerning the occurrence of this cestode in raccoon dogs in Poland is insufficient.

Among cestodes, several previous studies revealed infections with *Mesocestoides* spp. in raccoons and raccoon dogs [4,8,16,21,22]. The species of *Mesocestoides* Valliant, 1863 (Cyclophyllidea: Mesocestoididae) are cestodes that have been noted globally, other than in Australia and Antarctica. Definitive hosts are both wild and domestic terrestrial carnivores (Canidae, Mephitidae, Viverridae, Mustelidae, Procyonidae, Didephidae, Felidae and Hyaenidae), and rarely birds of prey [23]. *Mesocestoides* spp. have zoonotic potential, but records of infections in humans are sporadic [24]. Several species have been described based on morphology in the genus *Mesocestoides*, but the number of valid species and the use of morphological criteria for species identification have been questioned because of the lack of distinct morphological features, morphological variability within the species, and poorly defined actual host specificity and distribution over wide geographical regions have been noted [23,25,26]. So far, only four representatives of the genus—*Mesocestoides litteratus* (Batsch, 1786), *M. lineatus* (Goeze, 1782), *M. corti* (Hoeppli, 1925) (=*M. vogae* (Etges 1991) and *M. melesi* (Yanchev and Petrov, 1985), can reliably be identified as valid species, using both morphological and molecular characters [25,26,27]. 

In raccoon dogs, apart from the above-mentioned cestodes, *Dilepis undula*, *Dipylidium caninum* and *Taenia* spp. were recorded, mainly based on their morphological features [5,15,28]. There are several papers describing intestinal cestodes occurring in raccoons, most of them from North America [29,30], Japan [21] and others from Europe, including Poland [4,8,28]. To date, *P. lotor* has been reported as a natural definitive host of *Atriotaenia* spp. *Atriotaenia* Sandground, 1926, (Cestoda: Anoplocephalidae) has 5 valid species [31]. Three species have been reported from procyonids and bats in North and South America, i.e., *Atriotaenia procyonis* from the raccoon (*P. lotor*), *Atriotaenia sandgroundi* from the South American coati (*Nasua nasua*) and *Atriotaenia hastati* from the greater spear-nosed bat (*Phyllostomus hastatus*). Another species is described as parasitizing mustelids and procyonids from Europe and Asia, i.e., *Atriotaenia incisa* (Railliet, 1899) in the European badger (*Meles meles*) [31,32]. Priemer and Lux [32] did not find any morphological differences between *A. incisa* and *A. procyonis* and it is suggested that these species can be conspecific.

So far, to the best of our knowledge, the identification of cestodes in raccoons and raccoon dogs was often only solely based on morphological characters and parasites were classified to the genus level [8,33]. Therefore, the main aim of this research was to identify and estimate the prevalence of cestode species, as well as to compare the diversity of cestode species in definitive hosts in Poland (invasive carnivores, such as raccoons and raccoon dogs, and native carnivores, such as European badgers). Additionally, the study focused on the role of the raccoon dog in the epidemiology of *E. multilocularis* in Poland. Since methods based on the recovery of eggs from feces have poor sensitivity, and cestode eggs cannot be identified to species level based on morphology alone, we used PCR-based methods allowing the identification of tapeworms in carnivores.

## 2. Results

Overall, 55 of 261 (21.1%; 95% CI = 16.1–26.0%) fecal samples of carnivores were determined to be cestode-positive by PCR amplification of the region of the mt 12S rRNA gene using primers CES12sF and CES12sR (Table 1). Tapeworm DNA was detected in raccoons (19/103, 18.4%; 95% CI = 10.8–26.1%), raccoon dogs (13/111, 11.7%; 95% CI = 5.6–17.8%) and badgers (23/47, 48.9%; 95% CI = 34.1–63.8%). All amplification products were sequenced to provide an accurate identity by comparison to GenBank accessions. We did not find the DNA of *E. multilocularis* in the fecal samples of the raccoon dogs examined. Of the 55 amplified samples, 16 (29.1%) were classified to the genus *Mesocestoides*—*M. litteratus* (14.5%, 8/55), *M. lineatus* (3.6%, 2/55) and *M. melesi* (10.9%, 6/55) via the sequence homology search (Table 1). *Mesocestoides litteratus* was detected in both invasive carnivore species. 

**Figure 1:**
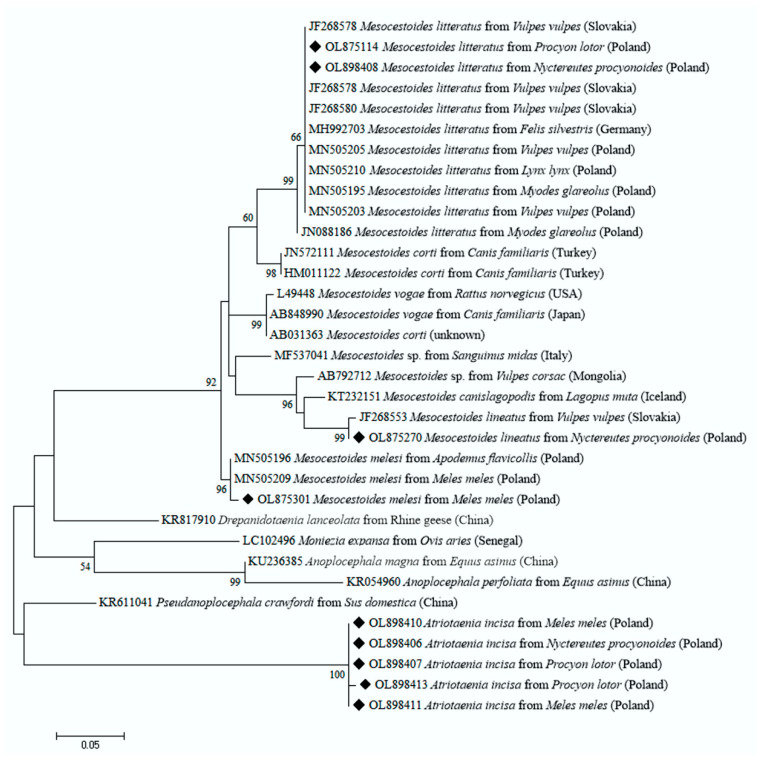
Maximum likelihood (ML) phylogenetic tree (GTR + G model) for *Mesocestoides* spp., *Atriotaenia* spp. and relatives based on sequences of the 12S rRNA gene fragment. Bootstrap values are shown above the branches. Each sequence is indicated by its tapeworm species GenBank accession number, host species and country of origin. The diamond signs indicate the sequences derived from this study. The dendrogram was constructed with 1000 replications using MEGA software.

All *M. litteratus* sequences obtained were identical to each other, and the BLAST analysis showed 100% similarity to those previously reported in other carnivores, including wild (i.e., *Felis silvestris* (MH992703)) and domestic species (i.e., *Canis lupus familiaris* (MH992710)), and 99.6% with sequence (MN505203) from *Vulpes vulpes.* On the other hand, *M. lineatus* was identified only in two samples derived from raccoon dogs, and the nucleotide sequences were 99.5% identical to *M. lineatus* sequence (JF268553) from *Vulpes vulpes* from Slovakia. Among *Mesocestoides* spp., we also identified *M. melesi* from a European badger. All of these sequences were identical to each other and the phylogenetic tree constructed based on the partial sequences of the mt 12S rRNA gene (Figure 1) showed that these sequences clustered together with *M. melesi* sequences derived from definitive and intermediate hosts from Poland (MN505196, MN505209). 

Interestingly, as many as 34 sequences obtained from *P. lotor, N. procyonoides* and *M. meles* were identical or almost identical (99.8%) to each other, but we found no match with any available sequences deposited in GenBank. This group of sequences displayed the highest similarity (only about 80%) to *M. vogae* (syn. *M. corti*) based on the sequences of the 12S rRNA gene fragment. Therefore, we collected adult cestodes (36 specimens) found during necropsy in the lumen of the small intestine of raccoons, as part of other studies [8]. Based on the morphological examinations and measurements, the samples were identified as *Atriotaenia incisa*. The main diagnostic features were: scolices measured up to 0.5 mm in width; the presence of four suckers, measuring 70–140 μm and situated in pairs on the surface of the scolex, but they also appeared to be withdrawn into the interior of the scolex in some cases; no hooks or other apical organs were present; the neck was short and the first proglottides were considerably broader than long [31,32,34,35]. Then, we analyzed by PCR and sequencing the adult cestodes obtained from the raccoon. The sequences of the 12S rRNA gene fragment derived from eight isolates of adult cestodes from the raccoons were identical to each other and identical or very similar (99.8%) to these 34 isolates from the feces samples from the raccoon, raccoon dog and European badger. The phylogenetic tree showed that these sequences created a separate clade, distant from all known species of cestodes available in the GenBank database (Figure 1). We were not able to identify another five isolates (four from *N. procyonoides*, one from *Meles meles*) at the species level because the sequence data of many species are still missing from the GenBank. 

## 3. Discussion

Invasive non-native species, such as raccoons and raccoon dogs, can act as definitive hosts of helminths, including tapeworms, which carry reproductive adults and shed large numbers of infectious eggs in the environment [36]. There are papers reporting intestinal cestodes occurring in raccoons and raccoon dogs, some of them from their native areas [29] and some from Europe [4,5,8,9]. To the best of our knowledge, the majority of studies concerning cestodes in these invasive carnivores (excluding those concerning *E. multilocularis* in raccoon dogs) were limited to adult worm morphological identification and classified only at the genus level [4,8]. In this study, the fecal samples of non-native invasive carnivores (i.e., raccoons and raccoon dogs) and, additionally, native carnivores (i.e., European badgers) were molecularly analyzed for the presence of cestode DNA. It is accepted that the parasite DNA excreted from eggs or proglottids can be detected from feces after amplification by PCR, and therefore copro-PCR can be used for confirmatory purposes for the identification of tapeworm eggs recovered from fecal samples [37]. In the present study, we investigated a fragment of mt 12S rRNA gene sequences. Currently, this is one of the accepted markers for the investigation of genetics and the characterization of cestode parasites, mainly because of its conserved structure, mode of inheritance and relatively high evolutionary rate [38].

In this research, based on the sequence analysis of the 12S rRNA gene marker, we confirmed the occurrence of tapeworms belonging to the genus *Mesocestoides*—*M. litteratus* in both invasive carnivore species, *M. lineatus* in raccoon dogs, and *M. melesi* in native carnivore species, the European badger. There is the first molecular evidence of these cestode species in invasive carnivores, additionally confirming their potential as reservoir hosts. Previous research concerning *Mesocestoides* spp. in raccoons and raccoon dogs essentially presented identification only at genus level. According to Jesudoss Chelladurai and Brewer [23], the global pooled prevalence rate for raccoons is 17.17% (95% CI: 10.66−24.85%), including studies from Asia (1 study; 531 samples; prevalence 0.2%), Europe (2 studies; 56 samples; prevalence 12.69%) and North America (15 studies; 2358 samples; prevalence 19.97%). In our study, the prevalence of *Mesocestoides* spp. is rather low, i.e., 1.9% in raccoons and 7.2% in raccoon dogs. In this last host species examined, the prevalence of *Mesocestoides* spp. was lower than that obtained in the Lithuanian (30.6% [15]) and Danish (23.2% [16]) surveys. On the other hand, in recent studies in Germany, no case of this helminth was recorded [22]. Our study supports the dominant occurrence of *M. litteratus* in carnivores from central Europe, in accordance with previous studies [25,39]. Nevertheless, we also identified *M. lineatus*, but only in raccoon dog samples and, to the best of our knowledge, this is the first molecular report of this cestode species in non-native invasive carnivores. Moreover, we molecularly confirmed the occurrence of *M. melesi* in badgers and our results are in line with the observations of Bajer et al. [27]. 

*Echinococcus multilocularis* DNA was absent from our study samples, suggesting that raccoon dogs can be considered unimportant as reservoir hosts for this parasite in Poland. This agrees with recent studies from Denmark [9] and Germany [22], although *E. multilocularis* is prevalent in raccoon dogs from other European countries, such as Lithuania and Latvia [12]. The absence of *E. multilocularis*-positive raccoon dogs in this study cannot exclude a negligible prevalence of these parasites in Polish raccoon dogs, especially since this cestode species is recorded with a high prevalence in red foxes in Poland [40].

In this research, taking into account both the morphological observations on adult cestodes derived from raccoons and the genetic analysis, we identified *A. incisa* in both the non-native invasive and native carnivores. Until now, this species has been identified only based on morphology in *P. lotor* and/or *M. meles* in Germany [32], Spain [41] and France [31]. Our report is the first to demonstrate the occurrence of this cestode species in *N. procyonoides.* In none of these reports has the parasite been described molecularly. Thus, in our study, *A. incisa* was for the first time molecularly characterized by using a fragment of mt 12S mt rRNA gene.

In conclusion, we have provided an analysis of the prevalence and diversity of cestodes in the wild populations of carnivores in Poland. Although we did not find the DNA of *E. multilocularis* in the fecal samples of the raccoon dogs examined, we proved the occurrence of the other zoonotic tapeworms, such as *Mesocestoides* spp. This research has contributed to a better understanding of the epidemiology of cestodes in carnivore hosts. 

## 4. Materials and Methods

### 4.1. Sample Collection

The material for this study, the fecal samples from two non-native invasive carnivore species (raccoon (n = 103) and raccoon dog (n = 111)) and, additionally, for comparative purposes, from the native European badger (n = 47) were collected from the large intestine of animals shot by hunters or found as roadkill in the area of western Poland, in Ruszów Forestry (51°24′00.1″ N, g15°10′12.2″ E) in the Lower Silesian District. Ruszów Forest District is located in the western part of the Lower Silesian Wilderness, the largest lowland forest complex in Europe. The intestines were kept in −80 °C for two weeks, for safety reasons, before examination [42]. Furthermore, we collected the adult individuals of cestodes (36 specimens) during the necropsy of the raccoons as part of other studies [8]. Adult tapeworms were found in the intestinal tracts, which had been frozen until dissection. The specimens were identified based on the general morphology, and the shape and size of scolex [31,32,34,35]. These cestode individuals were rinsed in PBS and thereafter preserved in 96% ethanol until further molecular studies.

### 4.2. DNA Extraction, PCR Amplification, Sequencing and Phylogenetic Analyses

The copro-DNA samples were extracted from feces using a Stool DNA Purification Kit (EURx, Gdansk, Poland). After the morphological identification of adult cestodes, DNA was isolated from eight of these specimens using a Bio-Trace DNA Purification Kit (EURx, Gdansk, Poland). The extracted copro-DNA and DNA from the adult cestode specimens were amplified using polymerase chain reaction by targeting a fragment of mitochondrial (mt) 12S rRNA gene using the primers CES12sF (5′-AGGGGATAGGACACAGTGCCAGC-3′) and CES12sR (5′-CGGTGTGTACMTGAGYTAAAC-3′) [43]. PCR reactions were performed in a T100 Thermal Cycler (Bio-Rad, Warsaw, Poland in a total volume of 25 µL containing 12.5 μL of 2 × PCR Mix Plus (A@A Biotechnology, Gdansk, Poland), 1.25 μL of each primer (10 mM), 4 μL of DNA template and 6 μL of ddH_2_O. PCR conditions included an initial denaturation step of 95 °C (3 min); 40 cycles of 95 °C (45 s), 55 °C (60 s) and 72 °C (60 s), and the final elongation of 72 °C (7 min). Additionally, due to the considerable public health importance of *E. multilocularis,* the fecal samples of the raccoon dogs were specifically analyzed by sensitive nested PCR for the amplified mt 12S rRNA gene fragments. The first reaction was performed using the primers p60for (5′-TTAAGATATATGTGGTACAGGATTAGATACCC-3′) and p375rev (5′-AACCGAGGGTGACGGGCGGTGTGTACC-3′) [44]. The second step PCR reactions were performed using a species-specific pair of primers, Em.nest/for (5′-GTGAGTGATTCTTGTTAGGGGAAGA-3′) and Em.nest/rev (5′-ACAATACCATATTACAACAATATTCCTATC-3′) [45]. Both reactions were conducted according to the protocol described by Lass et al. [46]. Negative controls containing distilled water instead of fecal DNA were run alongside PCRs to check for contamination. PCR products were separated by electrophoresis on a 1% agarose gel stained with SimplySafe (EURx, Gdansk, Poland). All the PCR-positive samples were purified and directly sequenced in both directions (Macrogen, Amsterdam, Netherlands) with the primers used for DNA amplification. The nucleotide sequences obtained in this study were manually edited by the use of the DNA Baser Sequence Assembly software v5.15.0 (Heracle BioSoft SRL Romania), and consensus sequences were aligned and compared with each other and with the corresponding sequences available in the GenBank database by BLASTn analysis (http://blast.ncbi.nlm.nih.gov/Blast.cgi, accessed on 18 December 2021). The alignments of the sequences were generated using the ClustalW algorithm implemented in MEGA 7.0. The phylogenetic tree was constructed using the Maximum Likelihood method with the MEGA 7.0 software [47]; bootstrapping was performed using 1000 replicates. 

The mt sequences of 12S rRNA gene obtained in this study were deposited in GenBank with the accession numbers OL875114; OL898408 for *M. litteratus*; OL875270 for *M. lineatus*; OL875301 for *M. melesi* and OL898406, OL898407, OL898410, OL898411 and OL898413 for *A. incisa*.

### 4.3. Statistics

We evaluated the prevalence of cestodes based on the positive PCR results of the stool samples. The 95% confidence interval (CI) was calculated for each prevalence.

## Figures and Tables

**Table 1:** Prevalence of the cestodes in raccoons, raccoon dogs and European badgers identified in this study.

Infecting Cestode	PCR Positive Fecal Samples/Number of Screened/(%; 95% CI)		
	Carnivore Species		
	*Procyon lotor*	*Nyctereutes procyonoides*	*Meles meles*
*Mesocestoides melesi*	0/103 (0)	0/111 (0)	6/47 (12.8; 95%CI 2.9–22.7)
*Mesocestoides litteratus*	2/103 (1.9; 95%CI 0–4.6)	6/111 (5.4; 95%CI 1.1–9.7)	0/47 (0)
*Mesocestoides lineatus*	0/103 (0)	2/111 (1.8; 95%CI 0–4.3)	0/47 (0)
*Atriotaenia incisa*	17/103 (16.5; 95%CI 9.2–23.8)	1/111 (0.9; 95%CI 0–2.7)	16/47 (34.0; 95%CI 20.0–48.1)
Unidentified cestode species	0/103 (0)	4/111 (3.6; 95%CI 0–7.1)	1/47 (2.1; 95%CI 0–6.4)

## Data Availability

The obtained nucleotide sequences were deposited in GenBank under the following accession numbers OL875114, OL898408, OL875270, OL875301, OL898406, OL898407, OL898410, OL898411 and OL898413.
